# 西达本胺在外周T细胞淋巴瘤维持治疗中的有效性及安全性

**DOI:** 10.3760/cma.j.cn121090-20240630-00238

**Published:** 2024-12

**Authors:** 华 尹, 金花 梁, 佳竹 吴, 悦 李, 馨予 张, 祎琳 孔, 必慧 潘, 莉 王, 建勇 李, 卫 徐, 浩睿 申

**Affiliations:** 南京医科大学第一附属医院（江苏省人民医院）血液科，南京 210029 Department of Hematology, the First Affiliated Hospital of Nanjing Medical University（Jiangsu Province Hospital）, Nanjing 210029, China

**Keywords:** 淋巴瘤，T细胞，外周, 西达本胺, 维持治疗, Lymphoma, T-cell, peripheral, Chidamide, Maintenance therapy

## Abstract

**目的:**

研究旨在评估西达本胺作为外周T细胞淋巴瘤（PTCL）一线或挽救治疗后口服药物维持的疗效和安全性。

**方法:**

回顾性分析2015年1月至2022年7月期间南京医科大学第一附属医院（江苏省人民医院）血液内科收治的58例PTCL患者的临床资料，所有患者均口服西达本胺作为一线或挽救治疗后的维持治疗。分析58例患者启动化疗后的无进展生存（PFS）期、总生存（OS）期以及评估西达本胺药物的安全性。

**结果:**

58例PTCL患者，男43例，女15例，中位年龄为66（29～83）岁。39例接受西达本胺作为一线维持治疗方案，19例患者接受西达本胺作为挽救性维持治疗方案。中位维持治疗时间16（1～72）个月，中位PFS期为33（2～74）个月，中位OS期未达到。接受西达本胺作为一线维持治疗方案的患者PFS和OS优于接受西达本胺作为挽救性维持治疗方案的患者（中位PFS期：未达到对7个月，*P*<0.001；中位OS期：未达到对67个月，*P*＝0.009）。最常见的不良反应是血液学不良反应（77.6％）。12例（20.7％）患者在治疗期间进行了剂量下调，3例患者因不良反应停止治疗。

**结论:**

西达本胺在PTCL的维持治疗中具有较好的疗效，特别是作为一线维持治疗具有较好的PFS和OS，且安全性良好。

## Abstract (en)

**Objective:**

This study aimed to assess the efficacy and safety of thioamide as a maintenance therapy for peripheral T-cell lymphoma（PTCL）.

**Methods:**

This study retrospectively analyzed the data from 58 patients with PTCL who were treated in the Department of Hematology at the First Affiliated Hospital of Nanjing Medical University from January 2015 to July 2022. Chidamide was orally administered as a maintenance therapy after first-line or salvage treatment. Progression-free survival（PFS）, overall survival（OS）, and safety were analyzed.

**Results:**

Among the 58 patients with PTCL, 43 were males and 15 were females, and the median age was 66（range: 29–83）years. Thirty-nine patients received thioamide as first-line maintenance therapy, and 19 patients received thioamide as maintenance therapy after salvage treatment. The median maintenance therapy duration was 16 months（range: 1–72 months）, with a median PFS time of 33（2–74）months, and the median OS time had not been reached. Patients who received first-line maintenance therapy with thioamide demonstrated superior PFS and OS outcomes compared with patients who received thioamide maintenance therapy after salvage treatment（median PFS time: not reached *vs* 7 months, *P*<0.001; median OS time: not reached *vs* 67 months, *P*＝0.009）. The most prevalent adverse reaction was a hematologic adverse reaction（77.6％）. Twelve（20.7％）patients underwent a dose reduction and three patients discontinued treatment.

**Conclusion:**

Patients receiving thioamide maintenance therapy demonstrated a promising PFS and OS with a manageable safety profile, especially as the first-line maintenance therapy.

外周T细胞淋巴瘤（PTCL）是非霍奇金淋巴瘤（NHL）中一组少见且具有高度异质性的肿瘤，预后较差[1]–[2]，中国的发病率远高于西方国家[3]。PTCL的常见病理类型包括PTCL-非特指型（PTCL-NOS）、血管免疫母细胞性T细胞淋巴瘤（AITL）、结外NK/T细胞淋巴瘤（ENKTL）、间变性大细胞淋巴瘤（ALCL）和单形性嗜上皮性肠T细胞淋巴瘤（MEITL）等[4]。目前PTCL尚无标准治疗方案，临床常用的化疗方案包括CHOP（环磷酰胺+阿霉素+长春新碱+泼尼松）方案或类CHOP方案，但总缓解率低、复发率高[5]–[8]。

组蛋白去乙酰化酶（HDAC）在肿瘤发生发展中发挥重要的作用，HDAC抑制剂（HDACi）可以抑制HDAC、促进染色质重塑、提高染色质组蛋白的乙酰化水平，并导致多种信号通路的表观遗传学发生变化，从而抑制肿瘤细胞周期、诱导肿瘤细胞凋亡，进而调节免疫活性、诱导和增强NK细胞和抗原特异性细胞毒性T淋巴细胞（CTL）介导的肿瘤杀伤效应[9]–[11]。罗米地辛、贝利司他以及西达本胺在治疗复发/难治性（R/R）PTCL中显示出疗效，并获批用于R/R PTCL的治疗[12]–[14]。然而，西达本胺在PTCL维持治疗中的作用及安全性仍需进一步探索。因此，本研究旨在评估西达本胺作为PTCL患者诱导治疗或挽救治疗后的维持治疗的有效性和安全性。

## 病例与方法

1. 病例：本研究为一项回顾性、单中心、单臂临床研究。回顾性分析了2015年1月至2022年7月期间南京医科大学第一附属医院江苏省人民医院血液内科收治的58例PTCL患者的临床资料，所有患者按照2016年世界卫生组织（WHO）淋巴细胞疾病分类诊断标准进行病理学诊断。纳入研究的患者均在诱导治疗或挽救治疗后达到完全缓解（CR）或部分缓解（PR）。本研究按照赫尔辛基宣言（2013年第64号）的原则进行。本研究已获得南京医科大学第一附属医院、江苏省人民医院集中伦理委员会的批准（批件号：2023-SR-297）。

2. 治疗方案：PTCL（除ENKTL外）患者的一线诱导治疗方案是以蒽环类药物为基础的联合化疗，对于不能耐受化疗的患者选择CPCT（西达本胺+泼尼松+环磷酰胺+沙利度胺）方案。ENKTL患者的一线诱导治疗方案是以门冬酰胺酶为基础的联合化疗。所有达到CR或PR的患者均进行auto-HSCT的可能性评估，无法行auto-HSCT的患者接受西达本胺维持治疗，具体为：每次口服20 mg或30 mg，每周2次［基于先前化疗后的骨髓抑制分级及其他不良事件（AE）调整用药剂量］。在维持治疗期间，通过临床检查和PET-CT或CT进行疗效评估，第一年每3个月评估1次，之后每6个月评估1次。根据2014版Lugano疗效评定标准[15]，分为CR、PR、疾病稳定（SD）和疾病进展（PD）。客观缓解率（ORR）为CR率+PR率。患者接受西达本胺维持治疗直至疾病进展或出现不可接受的不良反应。

3. 安全性评价：根据《常见不良反应事件评价标准（CTCAE）》5.0版进行AE分级。

4. 随访：通过住院病历、门诊就诊记录和电话对所有患者进行随访。截止随访时间为2023年6月，中位随访时间为39（2～79）个月。随访结局事件包括无进展生存（PFS）和总生存（OS）。OS期定义为从初始治疗至任何原因导致死亡或随访终止的时间。PFS期定义为从初始治疗至出现复发或进展的时间。缓解持续时间（DoR）定义为从肿瘤第1次评估为CR或PR开始到第1次评估为PD或死亡的时间。

5. 统计学处理：使用SPSS统计软件（版本26.0）、MedCalc统计软件（版本20.1.0）和Graphpad prism 9.0软件进行统计分析，分类变量以例数（％）表示，连续变量以*M*（范围）表示，组间比较采用*χ*^2^检验及Fisher确切概率法，采用Kaplan-Meier法计算PFS和OS，组间PFS和OS的比较采用Log-rank检验。*P*<0.05为差异有统计学意义。

## 结果

1. 临床特征：如表1所示，58例PTCL患者，男43例，女15例，中位年龄为66（29～83）岁。一线维持治疗的PTCL 39例，挽救性维持治疗的PTCL 19例。病理类型以AITL为主（58.6％），其次是PTCL-NOS（17.2％）、ALCL（13.8％）、ENKTL（8.6％）和MEITL（1.7％）。所有患者均接受西达本胺口服维持治疗，其中6例患者在auto-HSCT后继续维持，包括3例一线维持治疗和3例挽救性维持治疗的患者。

**表1:** 58例接受西达本胺维持治疗的外周T细胞淋巴瘤患者临床特征［例（％）］

临床特征	总体（58例）	一线维持治疗（39例）	挽救性维持治疗（19例）
男性	43（74.1）	29（74.4）	14（73.7）
年龄>60岁	39（67.2）	29（74.4）	10（52.6）
Lugano分期（Ⅲ～Ⅳ期）	34（58.6）	25（64.1）	9（47.4）
ECOG评分（2～4分）	6（10.0）	5（12.8）	1（5.3）
血清白蛋白<40 g/L	11（19.0）	9（23.1）	2（10.5）
IPI评分（3～5分）	28（48.3）	16（41.0）	12（63.2）
PIT评分（2～4分）	38（65.5）	24（61.5）	14（73.7）
病理分型			
AITL	34（58.6）	21（53.8）	13（68.4）
PTCL-NOS	10（17.2）	4（10.3）	6（31.6）
ALK^+^ ALCL	1（1.7）	1（2.6）	0（0）
ALK^-^ ALCL	7（12.1）	3（7.7）	4（21.1）
ENKTL	5（8.6）	1（2.6）	4（21.1）
MEITL	1（1.7）	1（2.6）	0（0）

**注** ECOG：美国东部肿瘤协作组；IPI：国际预后指数；PIT：T细胞淋巴瘤预后指数；AITL：血管免疫母细胞性T细胞淋巴瘤；PTCL-NOS：外周T细胞淋巴瘤-非特指型；ENKTL：结外NK/T细胞淋巴瘤；ALCL：间变性大细胞淋巴瘤；MEITL：单形性嗜上皮性肠T细胞淋巴瘤

一线维持治疗的患者中，30例患者接受了以蒽环类药物为基础的一线化疗、4例患者接受CPCT方案作为一线治疗、5例ENKTL患者接受了以门冬酰胺酶为基础的一线治疗。19例挽救性维持治疗的患者中，AITL 6例、PTCL-NOS 4例，ENKTL 4例、ALK^-^ ALCL 4例和MEITL 1例，既往系统治疗的中位数为2（2～4）次。所有挽救性维持治疗的患者均接受了包含西达本胺的联合化疗及后续维持治疗。

2. 疗效评价：58例患者中位随访时间为39（2～79）个月，中位PFS期为33（2～74）个月，中位OS期未达到（图1A、B）。2年的PFS率和OS率分别为57.8％和82.9％；接受西达本胺作为一线维持治疗的患者PFS期和OS期长于作为挽救性维持治疗的患者（中位PFS期：未达到对7个月，*P*<0.001；中位OS期：未达到对67个月，*P*＝0.009）（图1C、D）。AITL患者的中位PFS期未达到，PTCL-NOS、ENKTL、ALCL患者的中位PFS期分别为16.5（3～31）个月、41（5～46）个月、19（4～65）个月。AITL、PTCL-NOS和ENKTL患者的中位OS期未达到，而ALCL中位OS期为68（8～72）个月。不同病理亚型之间的PFS和OS差异均无统计学意义（均*P*>0.05，图1E、F）。

**图1:**
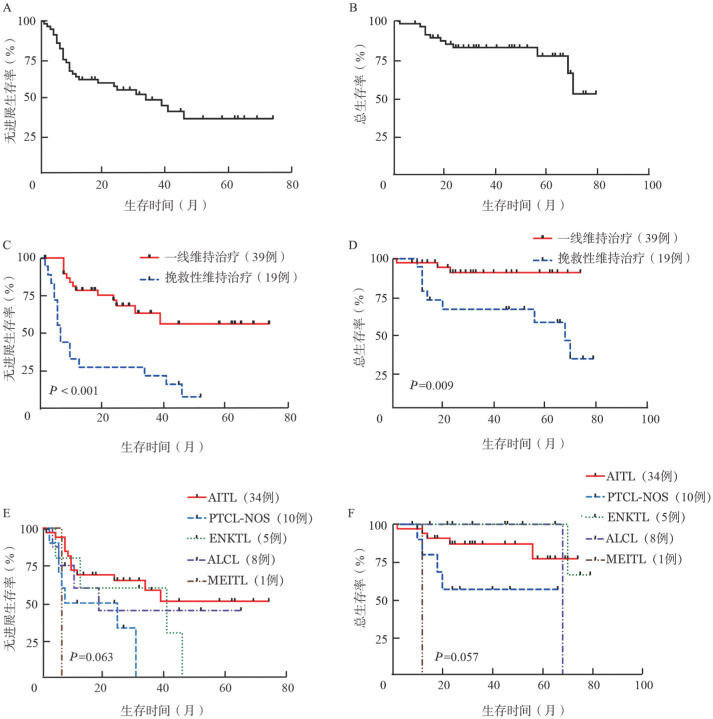
58例外周T细胞淋巴瘤患者进行西达本胺维持治疗的生存分析 **A** 58例患者无进展生存曲线；**B** 58例患者总生存曲线；**C** 西达本胺作为一线维持治疗及挽救性维持治疗的患者的无进展生存曲线；**D** 西达本胺作为一线维持治疗及挽救性维持治疗的患者的总生存曲线；**E** 不同病理亚型患者的无进展生存曲线；**F** 不同病理亚型患者的总生存曲线 **注** AITL：血管免疫母细胞性T细胞淋巴瘤；PTCL-NOS：外周T细胞淋巴瘤-非特指型；ENKTL：结外NK/T细胞淋巴瘤；ALCL：间变性大细胞淋巴瘤；MEITL：单形性嗜上皮性肠T细胞淋巴瘤

在39例一线维持治疗的患者中，有30例（76.9％）在CR后开始维持治疗，9例（23.1％）在达到PR后开始维持治疗。9例PR患者中有8例（88.9％）在维持治疗期间疗效评估达到了CR。11例（28.2％）患者出现PD，其中2例患者在因新型冠状病毒感染停药后2到3个月内出现PD。3例患者在auto-HSCT后继续行西达本胺维持治疗，2例患者持续CR，1例患者因个人原因1年后停止维持治疗，2个月后出现PD。

在19例挽救性维持治疗的PTCL患者中，9例（47.4％）PD，有9例（47.4％）在CR后接受了维持治疗，10例（52.6％）在达到PR后接受了维持治疗；其中有9例（47.4％）患者在维持治疗中出现PD，中位DoR为16（2～25）个月。3例患者在auto-HSCT后继续行西达本胺维持治疗，目前均持续CR，中位DoR为48（24～65）个月。

一线维持治疗和挽救性维持治疗的患者的中位DoR分别为26（4～79）个月和15（1～74）个月。截至末次随访日期，有29例患者的DoR超过24个月，8例患者的DoR超过60个月（表2）。单因素分析显示，患者的临床特征（如年龄、性别、国际预后指数、T细胞淋巴瘤预后指数、白蛋白、铁蛋白、β_2_微球蛋白或病理亚型）在PFS和OS中差异均无统计学意义（均*P*>0.05）。

**表2:** 缓解持续时间（DoR）超过60个月的外周T细胞淋巴瘤患者临床特征

例号	病理类型	年龄（岁）	性别	维持治疗	疾病分期（期）	IPI评分（分）	PIT评分（分）	维持治疗前的疾病状态	自体造血干细胞移植	OS期（月）	DoR（月）
1	AITL	76	女	一线	Ⅲ	3	3	CR	否	73	69
2	AITL	68	男	一线	Ⅳ	4	3	CR	否	68	63
3	AITL	56	男	一线	Ⅲ	2	2	CR	否	68	62
4	ALK^+^ ALCL	29	男	一线	Ⅳ	2	3	CR	否	73	65
5	AITL	65	女	一线	Ⅳ	3	3	CR	否	79	74
6	AITL	63	男	一线	Ⅲ	2	2	CR	是	68	62
7	PTCL-NOS	59	男	挽救性	Ⅲ	2	2	CR	否	74	66
8	AITL	61	女	挽救性	Ⅲ	2	2	CR	是	76	65

**注** IPI：国际预后指数；PIT：T细胞淋巴瘤预后指数；AITL：血管免疫母细胞性T细胞淋巴瘤；ALCL：间变性大细胞淋巴瘤；PTCL-NOS：外周T细胞淋巴瘤-非特指型；CR：完全缓解；OS：总生存

3. 安全性评价：所有患者的中位维持治疗时间为16（1～72）个月，中位维持剂量为20（15～30）mg，每周2次。最常见的不良反应是血液学不良反应（77.6％），包括中性粒细胞减少症（42例，72.4％）、贫血（23例，39.7％）和血小板减少症（29例，50.0％），其次是疲劳（35例，60.3％）、食欲减退（31例，53.4％）、肝功能异常（10例，17.2％）和肺炎（4例，6.9％）。大多数AE为1级或2级，3级至4级AE为中性粒细胞减少症（15/42）、血小板减少症（7/29）、疲劳（1/35）和肺炎（2/4）。12例（20.7％）患者因AE进行剂量减低，3例患者因AE停止治疗（其中2例为严重肺部感染，1例为无法忍受的疲劳）（表3）。1例因药物不可及在服用西达本胺10个月后停止了维持治疗，仍在接受随访。

**表3:** 58例接受西达本胺维持治疗的外周T细胞淋巴瘤患者的安全性［例（％）］

不良反应类型	出现不良反应	出现≥3级不良反应
血液学不良反应		
中性粒细胞减少	42（72.4）	15（25.9）
血小板减少	29（50.0）	7（12.1）
贫血	23（39.7）	0（0）
非血液学不良反应		
乏力	35（60.3）	1（1.7）
食欲减低	31（53.4）	0（0）
肺部感染	4（6.9）	2（3.4）
转氨酶升高（AST/ALT）	10（17.2）	0（0）

**注** AST：天冬氨酸氨基转移酶；ALT：丙氨酸氨基转移酶

## 讨论

PTCL异质性高且预后不良，ECHELON-2研究[16]显示维布妥昔单抗在CD30阳性的PTCL中有较好的疗效，且已被批准用于治疗CD30阳性的PTCL，但亚组分析显示仅ALCL患者获益显著，其他病理类型患者的PFS和OS没有显著改善。目前普遍认为auto-HSCT可以改善PTCL患者的预后，一项前瞻性多中心队列研究[17]显示，auto-HSCT组AITL患者的2年OS率和PFS率分别为93.3％和68.8％，而非auto-HSCT组则分别为52.9％和41.2％；但PTCL-NOS患者的中位OS期和PFS期在auto-HSCT组和非auto-HSCT组之间差异均无统计学意义（均*P*>0.05）；auto-HSCT在R/R PTCL中的作用仍未得到证实。与auto-HSCT相比，allo-HSCT可能产生更好的结果，但可能带来较高的非复发死亡率[18]。因此，探索不良反应小、能延长PTCL患者缓解时间的维持治疗方案亟待解决。

西达本胺是一种新型的口服HDACi，其可通过阻断细胞周期和调节凋亡蛋白抑制肿瘤细胞增殖、诱导细胞凋亡、激活NK细胞和抗原特异性CD8^+^ CTL介导的细胞抗肿瘤免疫，以及上调PTCL患者外周血PD-1^+^淋巴细胞细胞中的固有免疫相关基因从而发挥抗肿瘤效应[9]–[10],[19]，并在2014年被中国国家药品监督管理局批准用于治疗R/R PTCL[14]。基于西达本胺在治疗R/R PTCL中的良好疗效，多项临床研究进一步探索了西达本胺在新诊断PTCL患者中的疗效和安全性：一项针对中国人群的Ⅱ期临床研究[20]探索了CPET（西达本胺+泼尼松+依托泊苷+沙利度胺）方案在初诊未治的AITL患者中的疗效和安全性，结果显示，51例患者的ORR和CR率分别为90.2％和54.9％，中位PFS期为42.6个月，而中位OS期尚未达到。Wang等[21]比较了化疗联合西达本胺（ChT+C）与单独化疗（ChT）在新诊断PTCL患者中的疗效和安全性，结果显示ChT+C组患者的PFS优于ChT组患者，且在高IPI评分的患者中获益更多。上述研究表明，西达本胺联合化疗在PTCL的一线治疗中取得良好的疗效。

一项回顾性研究[22]对比了西达本胺联合CHOEP（西达本胺+环磷酰胺+阿霉素+长春新碱+泼尼松+依托泊苷）和CHOEP方案在初诊未治PTCL患者的疗效，发现西达本胺维持治疗呈现出提高3年PFS率（67.5％对54.2％）和OS率（87.5％对73.3％）的趋势。JACKPOT26研究[23]前瞻性探索了戈里昔替尼用于PTCL一线维持治疗的疗效及安全性。研究显示，30例诱导治疗后达CR的患者，中位随访时间为12个月，中位无病生存期未达到。18例诱导治疗后达PR的患者在维持治疗后，33％转为CR。因此，PTCL患者诱导治疗或挽救治疗后的维持治疗方案仍是我们需要探索的方向。

本研究评估西达本胺作为维持治疗在一线治疗或挽救治疗后的疗效和安全性。结果显示，接受一线维持治疗的患者比挽救治疗维持的患者有更好的PFS和OS，表明在一线治疗达到PR或CR后尽早使用西达本胺进行维持治疗可以降低复发率并延长PFS期和OS期，且安全性良好。值得注意的是，在一线治疗后达到PR的9例患者中有8例在维持治疗期间从PR转为CR（其中5例为AITL），表明西达本胺也可以进一步改善维持阶段的疗效，延长缓解时间。尽管不同病理亚型之间的PFS和OS差异无统计学意义，但AITL患者似乎有更好的PFS和OS。同时，在DoR超过60个月的8例患者中，有6例（75.0％）患者为AITL，其原因可能为AITL具有如TET2、RhoA和IDH2高突变的表观遗传学异常[24]–[25]，对于西达本胺的治疗获益更多。在本研究中有6例患者在一线或挽救治疗后接受了auto-HSCT，并后续接受了西达本胺维持治疗，1例患者在暂停维持治疗后PD，其余5例患者仍在缓解中。结果表明，在auto-HSCT后进行西达本胺维持治疗，可能使患者持续获益，但本研究由于例数较少且为回顾性研究，需进一步前瞻性临床研究证实。

综上，本研究结果显示西达本胺维持治疗可能改善PTCL患者的PFS及OS，尤其在一线治疗后进行维持治疗可能带来更大的生存获益，且安全性可控。由于本研究为单中心回顾性分析，样本量小，需前瞻性多中心研究且扩大样本量来验证西达本胺在PTCL中维持治疗的有效性和安全性。
